# Investigating the association between social participation and all-cause mortality risk among Chinese middle-aged and older adults

**DOI:** 10.3389/fpubh.2025.1596215

**Published:** 2025-07-15

**Authors:** Zhengxing Xu, Xuehui Gan, Jiaxin Zeng, Peijing Yan, Chao Yang

**Affiliations:** ^1^School of Public Health, Southwest Medical University, Luzhou, Sichuan, China; ^2^Department of General Surgery (Hepatobiliary Surgery), The Affiliated Hospital of Southwest Medical University, Luzhou, Sichuan, China; ^3^Clinical Research Center, Sichuan Provincial People's Hospital, University of Electronic Science and Technology of China, Chengdu, Sichuan, China

**Keywords:** social participation, all-cause mortality, middle-aged and older adults, prospective cohort study, China Health and Retirement Longitudinal Study

## Abstract

**Objective:**

While the association between social participation and all-cause mortality has been investigated to some extent, the link remains inconclusive, especially in China. This study aimed to investigate the association between social participation and all-cause mortality among middle-aged and older adult people in China.

**Methods:**

Using data from the China Health and Retirement Longitudinal Study, and altogether 15,883 participants were included. The Cox proportional hazards regression model was used to assess the relationship between social participation and all-cause mortality, and subgroup analyses were conducted by age (< 60 years, ≥ 60 years) and sex.

**Results:**

With a median follow-up of 9.01 years, 2,175 participants developed deaths. Results from multivariable Cox regression modeling showed participants who participated in social activities had an all-cause risk of death of 0.88 (*HR* = 0.88, 95% *CI*: 0.78, 0.99) compared with those who did not. Further analyses showed that compared with participants with no social participation, those with 1 item of social participation had an all-cause mortality risk of 0.93 (*HR* = 0.93, 95% *CI*: 0.81, 1.06), and those with ≥ 2 items were 0.77 (*HR* = 0.77, 95% *CI*: 0.64, 0.94). In addition, subgroup analyses showed no age (*P*_interaction_ = 0.571) or sex (*P*_interaction_ = 0.440) differences in the relationship between the both.

**Conclusion:**

Our results suggest social participation is an independent protective factor for all-cause death among middle-aged and older adult people in China. Active social participation helps to reduce the risk of death.

## 1 Introduction

With declining global mortality and fertility rates and increasing life expectancy, population aging has become a major medical and socio-demographic issue facing it worldwide (1). According to the World Health Organization estimates, one-sixth of the worldwide population will be over the age of 60 by 2030, and it will double to a staggering 2.1 billion in 2050 (1, 2). Considering the continuous increase in the global aging population, to achieve a successful transition from “active aging” to “healthy aging” (3), the factors affecting the health and longevity of older persons deserve to be widely explored.

Social participation is defined as “a person’s involvement in activities that provide interaction with others in society or the community” and is generally seen as an integral part of successful aging (4, 5). Participation in social or community activities can further positively impact an individual’s physical and psychological well-being by promoting a sense of belonging or purpose (5), which in part helps to reduce or slow down the occurrence of death.

Concerning the links between social participation and the risk of death, several previous epidemiologic studies have assessed the relationship to some extent, but have not produced entirely consistent results. Specifically, some studies have found that social participation is associated with the risk of all-cause mortality while reporting that social participation contributes to a lower risk of all-cause mortality (6–10). However, other studies have suggested no significant association between the both, especially after adjusting for potential confounders (11, 12). In addition, the evidence of existing studies mainly comes from developed countries such as Europe and the United States, and little is known about the relationship between social participation and the risk of all-cause mortality in China (12). To our knowledge, only two studies from China have so far explored the relationship using data from the Chinese Longitudinal Healthy Longevity Survey (13, 14). Specifically, one study investigated the association between social participation frequency (monthly or weekly engagement) and long-term survival time in older adults (13), while another examined the relationship between changes in social participation (transition from no participation to engagement) and all-cause mortality (14). Although both studies suggested that active social participation reduces all-cause mortality risk, neither directly explored the association between the presence/absence of social participation and all-cause mortality (13, 14). Therefore, given the incomplete consistency of existing global findings and the limitations of existing studies in China, it is essential that new research be conducted to statistically measure the relationship between social participation and mortality risk.

Therefore, leveraging longitudinal survey data derived from the China Health and Retirement Longitudinal Study (CHARLS) (15), this study attempted to assess the potential prospective association between social activity participation and all-cause mortality among middle-aged and older adults in China.

## 2 Materials and methods

### 2.1 Study population and sample

We use data from CHARLS,^1^ a nationally representative longitudinal follow-up survey of individuals aged 45 years and older and their spouses in China. Detailed information related to the study design has been reported elsewhere (15). Concerning data collection, CHARLS applies a variety of methods to collect data, including face-to-face interviews to collect questionnaire data, physical examinations to obtain body measurements, and collection of blood samples to obtain biomarker data. Of these, for the collection of questionnaire data, the specific practice was for trained interviewers to use Computer Assisted Personal Interviewing to conduct face-to-face surveys, systematically presenting questions through a computer program in accordance with the questionnaire’s logical structure and recording respondents’ answers directly into the system. Following each data collection wave, CHARLS compiles and publicly releases complete survey data, to date, five waves of survey data have been publicly released. The CHARLS program was approved by the Biomedical Ethics Committee of Peking University (No. IRB00001052–11015) for all baseline and follow-up surveys and all participants signed an informed consent form before data collection.

**Figure 1:**
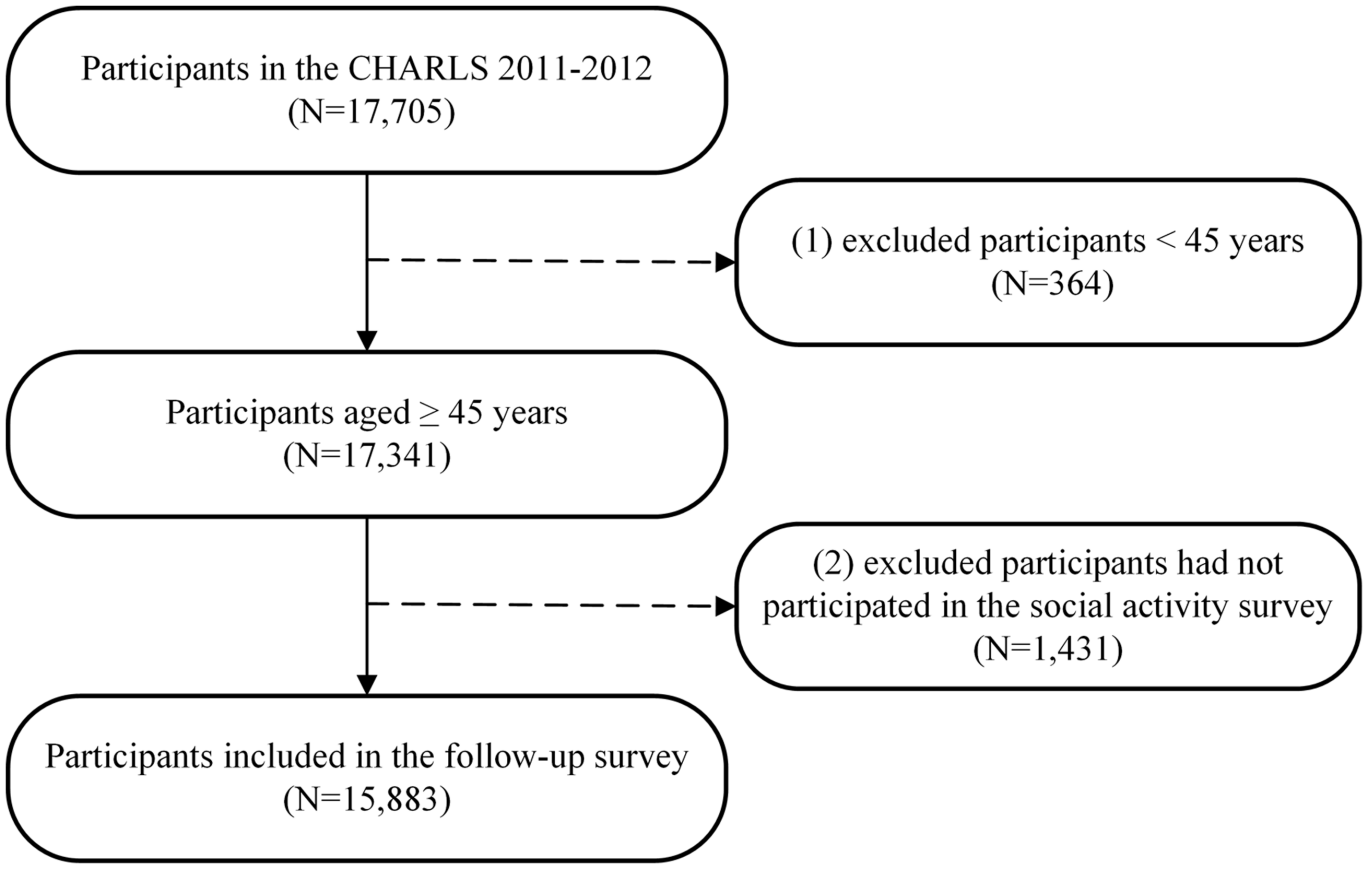
Selection process of participants in this study.

In this study, we utilized data from five waves of CHARLS surveys in 2011 (baseline survey), 2013 (first wave of follow-up), 2015 (second wave of follow-up), 2018 (third wave of follow-up), and 2020 (fourth wave of follow-up) for analysis. Specifically, a total of 17,705 participants were enrolled in the baseline survey in 2011. We initially excluded participants aged < 45 years (*n* = 364) and those who did not participate in the social activity survey at baseline (*n* = 1,431), resulting in a final sample of 15,883 participants for the follow-up survey (Figure 1). During the follow-up period, a total of 1,509 participants (9.50%) were lost to follow-up. A comparison of the characteristics of those who were lost during follow-up with those who were not is shown in Supplementary Table S1.

### 2.2 Assessment of social participation

Social participation was assessed by asking participants the following equally weighted question: “Have you done any of these activities in the last month” (16). The specific social activities include: (1) Interacted with friends; (2) Played Ma-jong, played chess, played cards, or went to community club; (3) Provided help to family, friends, or neighbors who do not live with you and who did not pay you for the help; (4) Went to a sport, social, or other kind of club; (5) Took part in a community-related organization; (6) Done voluntary or charity work; (7) Cared for a sick or disabled adult who does not live with you and who did not pay you for the help; (8) Attended an educational or training course; (9) Stock investment; (10) Used the Internet and (11) engaged in other activities. The numbers of participants in each social activity are detailed in Supplementary Table S2. For each participant, we calculated the total number of social participations by cumulatively summing the assigned values for the 11 activities described above. If the total number of social participations is 0, it means that the participant did not engage in social participation; conversely, a total of ≥ 1 means that the participant did engage in social participation (16, 17). To assess the effect of the level of social participation on all-cause mortality, we further categorized the total number of social participations into three categories based on previous literature, that is, totals of 0, 1, and ≥ 2 (7, 16).

### 2.3 Assessment of all-cause deaths

Our interest outcome was all-cause death, which was determined based on the interview status (alive or dead) of the participants at the time of each follow-up survey (18, 19). Simultaneously, we also collected the survival time of the participants in the study. It is important to note that in the CHARLS dataset, the exact time of death of participants was only recorded in the Wave 1 Follow-up Survey and Wave 4 Follow-up Survey. If a precise time was recorded at the time of the participant’s death event, survival time was calculated as the interval from the date of the baseline investigation to the exact date of the participant’s death; Conversely, if the exact time to the death event was not available, survival time was estimated based on the median time from the date of the baseline survey to the wave in which the death was recorded (18, 19).

### 2.4 Measurement of covariates

Based on previous literature (20, 21), we collected information on several covariates to adjust for confounders (Supplementary Table S3). The depressive symptom was measured using the 10-item Center for Epidemiologic Studies Depression Scale (CES-D) (22), which was categorized as with (CES-D ≥ 10 scores) or without (CES-D < 10 scores) depressive symptom based on depressive symptom score (23). Cognitive function was assessed using the Chinese version of the Mini-Mental State Examination (MMSE) for two related domains of cognitive function (situational memory and mental integrity) and was categorized as either cognitively impaired (MMSE < 11) or cognitively intact (MMSE ≥ 11) based on MMSE scores (24). The Activities of Daily Living (ADL) were assessed using the ADL scale, which covers 11 domains: eating, bathing, dressing, indoor moving, toileting, continence control, performing housework, shopping, cooking, taking medicine, and financial management (25). ADL disability was determined based on whether an individual is unable or has difficulty completing any of the 11 items (25). Physical frailty was measured using a modified version of the Fried physical frailty phenotype approach, which consists of five dimensions: shrinking, weakness, slowness, low physical activity, and exhaustion (26). If participants met three or more criteria they were defined as with and without physical frailty (27). Hypertension was defined as participant self-reported hypertension diagnosed by doctors, using antihypertensive medication, systolic blood pressure ≥ 140 mmHg, or diastolic blood pressure ≥ 90 mmHg (28). Diabetes mellitus was defined as participant self-reported diabetes diagnosed by doctors, use of anti-diabetic medications (including use of insulin or oral hypoglycemic agents), fasting blood glucose ≥ 126 mg/dL, or glycosylated hemoglobin ≥ 6.5% (29). Heart disease, stroke, and cancer were determined based on participants’ self-reported physician diagnoses.

### 2.5 Statistical analyses

The normal distribution is expressed as mean ± standard deviation for continuous variables, median (interquartile range) for non-normally distributed continuous variables, and frequency (percentage) for categorical variables. The group comparisons of baseline characteristics were compared using the *t*-test or Mann–Whitney *U* test for continuous variables and the *χ^2^*-test for categorical variables.

Person-years of follow-up were calculated for each participant from the date of the baseline survey until the date of the occurrence of the endpoint event (death), loss of follow-up, or end of follow-up, whichever came first. The Cox proportional hazards model with hazard ratios (*HR*s) and corresponding 95% confidence intervals (*CI*s) was employed to assess the relationship between social participation and all-cause mortality. Three models were applied to minimize the role of potential confounders, including model 0 unadjusted for any covariates, model 1 adjusted only for age and sex, and model 2 based on model 1 further adjusted for the place of residence, marital status, educational attainment, annual household living expenditures, smoking status, alcohol consumption status, depressive symptom, cognitive function, physical frailty, disability in activities of daily living, hypertension, diabetes mellitus, heart disease, stroke, and cancer. To further assess the relationship between the level of social participation and the risk of all-cause mortality, the amount of social participation was converted to a three-categorical variable by 0, 1, and ≥ 2 for inclusion in the Cox model and tested for a trend. Meanwhile, the restricted cubic spline (RCS) regression model was used to assess the potential nonlinear association between social participation and all-cause mortality using the 10th, 50th, and 90th percentiles of amounts of social participation as nodes and the 10th percentile as reference (30).

In addition, considering that age and sex characteristics may be influencing the association between social participation and all-cause mortality, subgroup analyses were conducted by age (< 60 years, ≥ 60 years) and sex (male, female) (16), with potential interactions assessed by adding interaction terms. Finally, two sensitivity analyses were carried out to assess the robustness of the primary results. First, to minimize potential reverse causality, we repeated the main analysis after excluding participants with less than 2 years of follow-up (31). Second, to fully utilize the available sample data, we performed multiple imputations of chained equations (MICE) for data missing covariate characteristics and repeated the main analysis using the dataset with multiple imputations (32, 33).

All the above analyses were performed using R version 4.0.5. All the hypothesis tests were performed using two-sided tests, and *P* values less than or equal to 0.05 were considered statistically different.

## 3 Results

### 3.1 Baseline characteristics of the participants

A total of 15,883 participants, a median age was 58 (52, 66) years, and a percentage of males 47.66%, were included in the final analysis sample of this study (Table 1). There were 8,027 (51.67%) participants in the study with social participation at the time of the baseline survey. In comparison to the participants of the study who were socially engaged and not socially engaged, a statistically significant difference was observed in age, gender, place of residence, education, annual household living expenditure, smoking status, alcohol consumption status, depressive symptom, cognitive function, physical frailty, disability in activities of daily living and diabetes mellitus characteristics (*P* < 0.05).

**Table 1:** The baseline characteristics of the participants in the study.

Characteristics	Total sample (*N* = 15,883)	Social participation		*P*-value ^a^
		No (*N* = 7,856)	Yes (*N* = 8,027)	
Age, years	58 (52, 66)	59 (53, 66)	58 (51, 65)	< 0.001
Sex				0.040
Male	7,565 (47.66)	3,676 (46.83)	3,889 (48.47)	
Female	8,308 (52.34)	4,174 (53.17)	4,134 (51.53)	
Place of residence				< 0.001
Village	12,144 (76.57)	6,334 (80.76)	5,810 (72.48)	
Urban	3,715 (23.43)	1,509 (19.24)	2,206 (27.52)	
Educational attainment				< 0.001
Primary school and lower	10,634 (66.99)	5,699 (72.60)	4,935 (61.50)	
Junior middle school and higher	5,241 (33.01)	2,151 (27.40)	3,090 (38.50)	
Marital status				0.087
Married	13,837 (87.12)	6,807 (86.66)	7,030 (87.58)	
Other ^b^	2,045 (12.88)	1,048 (13.34)	997 (12.42)	
Annual household living expenditure, RMB	7,280 (3,640, 14,040)	6,500 (3,120, 12,844)	7,800 (3,640, 15,600)	< 0.001
Smoking status				< 0.001
Never	9,642 (60.73)	4,910 (62.51)	4,732 (58.99)	
Formal	1,410 (8.88)	670 (8.53)	740 (9.22)	
Current	4,825 (30.39)	2,275 (28.96)	2,550 (31.79)	
Alcohol consumption status				< 0.001
Never	10,699 (67.38)	5,519 (70.26)	5,180 (64.56)	
Drinking but less than once a month	1,220 (7.68)	496 (6.31)	724 (9.02)	
Drinking more than once a month	3,959 (24.93)	1,840 (23.42)	2,119 (26.41)	
Depressive symptom				< 0.001
No	9,410 (62.96)	4,254 (58.22)	5,156 (67.50)	
Yes	5,535 (37.04)	3,053 (41.78)	2,482 (32.50)	
Cognitive function				< 0.001
Intact	8,743 (81.97)	3,759 (77.04)	4,984 (86.12)	
Impairment	1,923 (18.03)	1,120 (22.96)	803 (13.88)	
Physical frailty				< 0.001
No	12,565 (93.02)	6,117 (91.63)	6,448 (94.38)	
Yes	943 (6.98)	559 (8.37)	384 (5.62)	
Disability in activities of daily living				< 0.001
No	13,566 (87.50)	6,428 (82.12)	7,138 (89.21)	
Yes	2,263 (14.30)	1,400 (17.88)	863 (10.79)	
Hypertension				0.386
No	9,764 (61.70)	4,859 (62.05)	4,905 (61.37)	
Yes	6,060 (38.30)	2,972 (37.95)	3,088 (38.63)	
Diabetes mellitus				0.013
No	13,590 (86.29)	6,786 (86.98)	6,804 (85.61)	
Yes	2,160 (13.71)	1,016 (13.02)	1,144 (14.39)	
Heart disease				0.915
No	13,839 (87.63)	6,848 (87.59)	6,991 (87.66)	
Yes	1,954 (12.37)	970 (12.41)	984 (12.34)	
Stroke				0.089
No	15,484 (97.73)	7,642 (97.52)	7,842 (97.94)	
Yes	359 (2.27)	194 (2.48)	165 (2.06)	
Cancer				0.953
No	15,648 (98.98)	7,739 (98.96)	7,909 (98.99)	
Yes	162 (1.02)	81 (1.04)	81 (1.01)	

Data are frequency (%) for categorical variables and median (interquartile range) for continuous variables. ^a^Group comparisons of baseline characteristics were compared using the Mann–Whitney U test for continuous variables and the χ^2^-test for categorical variables. ^b^Other includes separated, divorced, widowed, and never married.

### 3.2 Association between social participation and all-cause mortality

The median follow-up of the participants in the study was 9.01 (8.67, 9.01) years, with a cumulative follow-up of 128,789 person-years, and deaths occurred in a total of 2,175 participants (Table 2 and Supplementary Tables S4, S5). Among them, the participants without social participation at baseline developed 1,205 deaths with an incidence density of 18.99/1,000 person-years, while those with social participation at baseline developed 970 deaths with an incidence density of 14.84/1,000 person-years. After adjusting for all potential confounders, results from Cox proportional hazards regression modeling showed that participants who participated in social activities at baseline had an all-cause risk of death of 0.88 (*HR* = 0.88, 95% *CI*: 0.78, 0.99) compared with participants who did not participate in social activities at baseline.

**Table 2:** The prospective association between social participation and all-cause mortality.

Exposure	Cases/person-years	*HR* (95% *CI*)		
		Model 0^a^	Model 1^b^	Model 2^c^
Social participation (dichotomy)				
No	1,205/63,443	1.00 (reference)	1.00 (reference)	1.00 (reference)
Yes	970/65,346	0.78 (0.72, 0.85) ***	0.84 (0.77, 0.91) ***	0.88 (0.78, 0.99) *
Social participation (three categories)				
0	1,205/63,443	1.00 (reference)	1.00 (reference)	1.00 (reference)
1	737/43,187	0.90 (0.82, 0.98) *	0.91 (0.83, 1.00) *	0.93 (0.81, 1.06)
≥ 2	233/22,159	0.55 (0.48, 0.63) ***	0.68 (0.59, 0.78) ***	0.77 (0.64, 0.94) **
*P* for trend	–	< 0.001	< 0.001	0.011
Social participation (continuous)	2,175/128,789	0.79 (0.75, 0.84) ***	0.85 (0.81, 0.90) ***	0.89 (0.82, 0.96) **

^a^Model 0 was not adjusted for any covariate. ^b^Model 1 adjusted only for age and sex. ^c^Model 2 further adjusts for the place of residence, marital status, educational attainment, annual household living expenditures, smoking status, alcohol consumption status, depressive symptom, cognitive function, physical frailty, disability in activities of daily living, hypertension, diabetes mellitus, heart disease, stroke, and cancer based on Model 1. ^*^ *P* ≤ 0.05; ^**^ *P* ≤ 0.01; ^***^ *P* ≤ 0.001. *HR*, Hazard ratio; *CI*, Confidence interval.

Further assessing the relationship between the extent of social participation and all-cause mortality (Table 2 and Supplementary Table S6) showed that after adjusting for all potential confounders, compared with participants with no social participation, those with 1 item of social participation had an all-cause mortality risk of 0.93 (*HR* = 0.93, 95% *CI*: 0.81, 1.06), and those with ≥ 2 items of social participation was 0.77 (*HR* = 0.77, 95% *CI*: 0.64, 0.94). The results of the test for trend showed that the participant’s risk of all-cause mortality decreases progressively as the extent of social participation increases (*P* for trend = 0.011).

**Figure 2:**
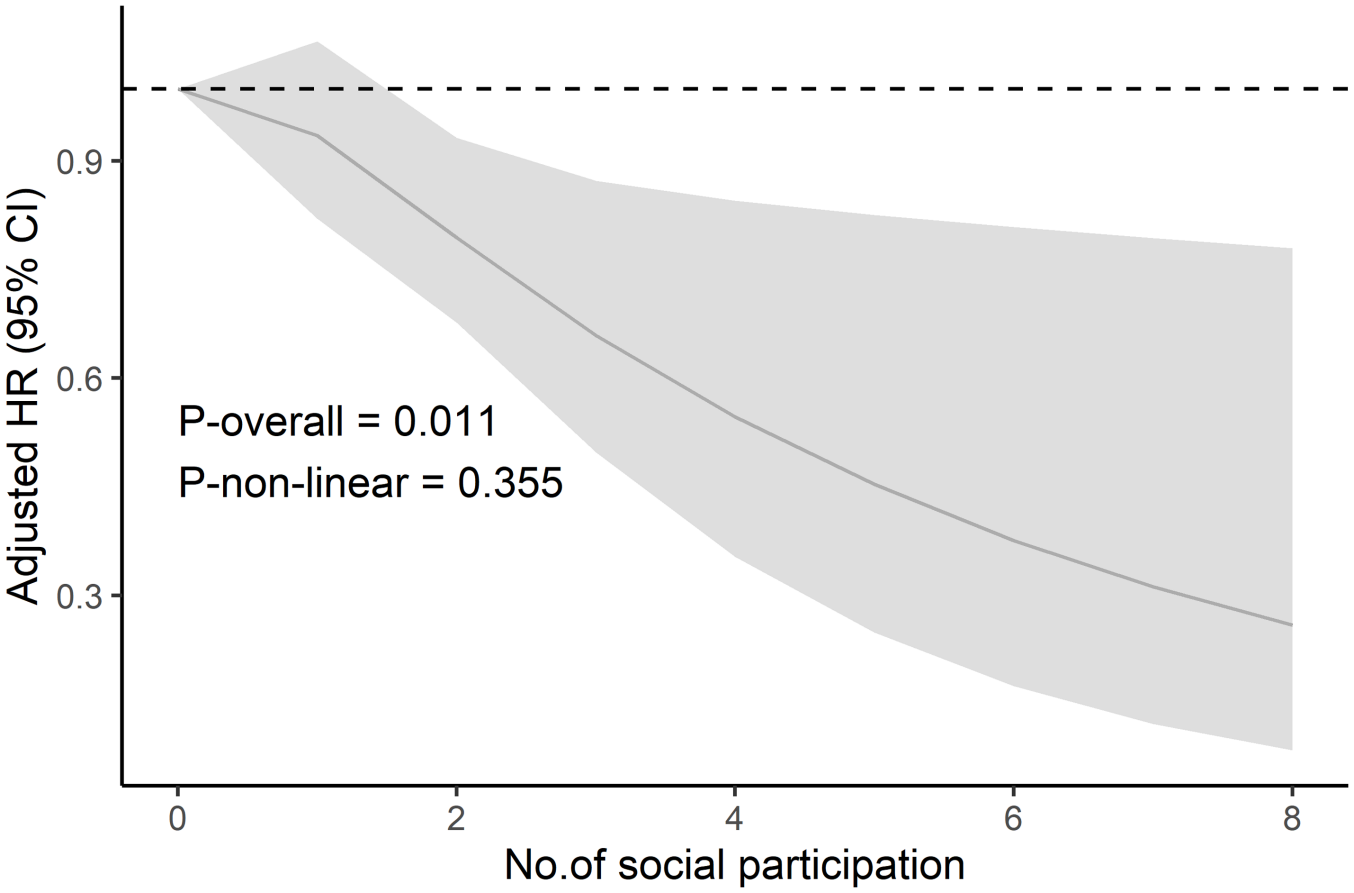
The restricted cubic spline curve of social participation and risk of all-cause mortality. The restricted cubic spline model adjusted for age, gender, the place of residence, marital status, educational attainment, annual household living expenditures, smoking status, alcohol consumption status, depressive symptom, cognitive function, physical frailty, disability in activities of daily living, hypertension, diabetes mellitus, heart disease, stroke, and cancer. Abbreviations: *HR* = Hazard ratio, *CI* = Confidence interval.

In addition, an RCS regression model was used to assess the nonlinear relationship between social participation and all-cause mortality (Figure 2), which showed that there was insufficient evidence to support a nonlinear relationship between the amount of social participation and all-cause mortality after adjusting for all potential confounders (*P*_nonlinear_ = 0.355).

### 3.3 Subgroup analysis

The results of subgroup analyses by age showed that after adjusting for all potential confounders, the association between social participation and the risk of all-cause mortality was statistically significant only among those ≥ 60 years of age (*HR* = 0.88, 95% *CI*: 0.80, 0.97), but no significant effect modification was observed (*P*_interaction_ = 0.571). Similarly, the analysis of the results by sex subgroup showed that the association between social participation and risk of all-cause mortality was statistically significant in both males (*HR* = 0.90, 95% *CI*: 0.82, 0.98) and females (*HR* = 0.84, 95% *CI*: 0.72, 0.99), but no significant effect modification was observed either (*P*_interaction_ = 0.440) (Table 3).

**Table 3:** Age and sex subgroup analysis of the association between social participation and risk of all-cause mortality.

Subgroup	Cases/person-years	HR (95% CI)			*P* interaction^d^
		Model 0^a^	Model 1^b^	Model 2^c^	
Age					0.571
< 60 years (*N* = 8,757)	440/73,043	0.89 (0.80, 0.99) *	0.87 (0.79, 0.97) *	0.91 (0.79, 1.05)	
≥ 60 years (*N* = 7,126)	1,735/55,746	0.83 (0.78, 0.88) ***	0.82 (0.77, 0.87) ***	0.88 (0.80, 0.97) **	
Sex					0.440
Male (*N* = 7,565)	1,289/60,739	0.79 (0.74, 0.84) ***	0.85 (0.79, 0.91) ***	0.90 (0.82, 0.98) *	
Female (*N* = 8,308)	884/67,969	0.79 (0.72, 0.86) ***	0.86 (0.78, 0.94) **	0.84 (0.72, 0.99) *	

^a^Model 0 was not adjusted for any covariate. ^b^Model 1 adjusted only for age and sex, but the subgroup factors themselves were not adjusted in the different subgroups. ^c^Model 2 was adjusted for age, gender, place of residence, marital status, educational attainment, annual household living expenditures, smoking status, alcohol consumption status, depressive symptom, cognitive function, physical frailty, disability in activities of daily living, hypertension, diabetes mellitus, heart disease, stroke, and cancer but the subgroup factors themselves were not adjusted in the different subgroups. ^d^Interaction analysis was performed only for model 2. ^*^P ≤ 0.05; ^**^P ≤ 0.01; ^***^P ≤ 0.001. *HR*, Hazard ratio; *CI*, Confidence interval.

### 3.4 Sensitivity analysis

The results of the two sensitivity analyses showed that both sensitivity analysis 1 (excluded participants with less than 2 years of follow-up) and sensitivity analysis 2 (utilized the multiple imputations dataset) presented results that remained generally consistent with the main results (Table 2) in terms of the magnitude and direction of the association effect, suggesting that our results are robust (Supplementary Table S7).

## 4 Discussion

By utilizing data from the nationally representative CHARLS, we assessed longitudinally the association between social participation and the risk of all-cause mortality among middle-aged and older adults in China. Our results show that social participation is associated with a reduced risk of all-cause mortality in middle-aged and older adult Chinese and that the risk of all-cause mortality decreases progressively as the amount of social participation increases.

Our study found that social participation was associated with a reduced risk of all-cause mortality, which is consistent with the findings of several previous studies (6–8, 10). For instance, a prospective cohort study from Chile found that participants with social participation reduced the risk of all-cause mortality by 22% (*HR* = 0.78, 95% *CI*: 0.63, 0.95) compared to those without social participation (10). In addition, previous studies have also hinted that a greater quantity of social participation was associated with lower all-cause mortality, but the results are inconclusive (7). For the present study, we found that participants who reported participation in two or more (*HR* = 0.77, 95% *CI*: 0.64, 0.94) social activities had lower all-cause mortality rates than those who participated in only one (*HR* = 0.93, 95% *CI*: 0.81, 1.06) or none, with a trend toward progressively decreasing rates (*P* for trend = 0.011), which somewhat confirms the findings of previous studies (7). Simultaneously, it also suggested that there may be a threshold effect in the relationship between social participation and the risk of all-cause mortality and that only a sufficient amount of social participation can be beneficial to the health of middle-aged and older adults.

Several potential mechanisms could help explain the relationship between social participation and reduced risk of all-cause mortality. On the one hand, social participation can influence all-cause mortality by promoting individual health behaviors. Previous studies have found positive associations between social participation and health behaviors, including dropping risky health behaviors (e.g., smoking cessation) (34) and engaging in beneficial health behaviors (e.g., engaging in physical activity during leisure time, maintaining an adequate amount of sleep, or increasing intake of fruits and vegetables) (35, 36). These health behaviors are established protective factors for influencing death. Hence, health behaviors would mediate the relationship between social engagement and all-cause mortality, resulting in lower all-cause mortality. On the other hand, social participation can also protect physical health by having a positive impact on mental health or psychological states (e.g., lower levels of depressive symptoms) (37). Although these potential mechanisms contribute to explaining the relationship between the two, the health behavior and mental health pathways discussed above represent theoretical explanations derived from synthesizing inductive insights in the existing literature. As such, future research is still warranted to employ mediation analysis for further validation of these hypothesized mechanisms.

In addition, a few previous studies have found that there may be age and sex differences in the association between social participation and health risks (12, 16, 38). Concerning age, compared to older people, other age groups are more likely or have more channels to engage in social activities and would therefore benefit more from them (12). For gender, the fact that women have higher family responsibilities and spend more time at home can make their health more sensitive to social participation outside the home (16). Consequently, social participation may have a greater impact on females than males. However, in the current study, we did not observe a significant age and sex effect modifier for the association between social participation and risk of all-cause mortality, although it was more sensitive in ≥ 60 years and in female participants, which is not entirely consistent with previous findings. Future studies need to pay further attention to the specificity of the association.

Some limitations of our study need to be acknowledged. First, the assessment of social participation is based on self-reporting by the participants, and recall bias is inevitable, which may lead to misestimation of the results. Second, the status of social participation of participants was assessed only at the baseline survey, and we did not consider the effect of changes in social participation over time during follow-up on all-cause mortality. Third, although our study adjusted for a subset of important confounders in the multifactor model, the effect on the results of some other residual confounding that was not measured or not accurately measured cannot be ruled out. Fourth, cultural disparities across nations may lead to differences in the common types of social participation among study participants. For instance, the primary form of social participation identified in this study was interaction with friends (Supplementary Table S2), whereas a study from Japan suggested that the common type was participating in the neighborhood communities (8). Such cross-cultural variations in social participation types may impose potential limitations on generalizing the study’s findings to populations in other countries. Given the limitations of the current study, these results need to be interpreted with caution, and further investigations are needed to support our findings.

In conclusion, based on this longitudinal study, our results suggest that social participation is an independent protective factor for all-cause death in middle-aged and older Chinese adults and that active social participation contributes to the reduction of death risk. Considering the increasing trend of global aging, our evidence emphasizes that middle-aged and older adult people should be encouraged to participate more in social activities in various ways to enhance their health.

## Data Availability

The original contributions presented in the study are included in the article/Supplementary material, further inquiries can be directed to the corresponding authors.

## Footnotes

^1^ https://charls.pku.edu.cn/
