# Supplementary material for: Investigating the association between social participation and all-cause mortality risk among Chinese middle-aged and older adults
Source: Front Public Health. 2025 Jul 15;13:1596215. doi: 10.3389/fpubh.2025.1596215 (PMC12303972; doi:10.3389/fpubh.2025.1596215)
Supplement: Supplementary file 1 [file Table_1.DOCX]

***Supplementary material***

**Supplementary Tables**

**Supplementary Table S1**. The comparison of the characteristics of lost and non-lost to follow-up participants.

**Supplementary Table S2**. The number of study participants in various social activities.

**Supplementary Table S3**. Description of assignment of covariate characteristics.

**Supplementary Table S4**. The prospective association between social participation (continuous) and all-cause mortality.

**Supplementary Table S5**. The prospective association between social participation (dichotomy) and all-cause mortality.

**Supplementary Table S6**. The prospective association between social participation (three categories) and all-cause mortality.

**Supplementary Table S7**. Sensitivity analysis.

**Supplementary Table S1**. The comparison of the characteristics of lost and non-lost to follow-up participants.

| Characteristics | Loss to follow-up | | *P value ^a^* |
| --- | --- | --- | --- |
|  | No  (N = 14,374) | Yes  (N = 1,509) |  |
| Age (years), Median (P25, P75) | 58 (52, 66) | 57 (50, 65) | < 0.001 |
| Male, n (%) | 6,871 (47.83) | 694 (46.02) | 0.190 |
| Village, n (%) | 11,477 (79.96) | 667 (44.29) | < 0.001 |
| Primary school and lower, n (%) | 9,831 (68.42) | 803 (53.28) | < 0.001 |
| Marital status, n (%) | 12,524 (87.13) | 1,313 (87.07) | 0.979 |
| Annual household living expenditure (RMB), Median (P25, P75) | 7,280  (3,432, 13,520) | 10,400  (5,200, 16,484) | < 0.001 |
| Smoking status, n (%) |  |  | 0.006 |
| Never | 8,688 (60.47) | 954 (63.22) |  |
| Formal | 1,261 (8.78) | 149 (9.87) |  |
| Current | 4,419 (30.76) | 406 (26.91) |  |
| Alcohol consumption status, n (%) |  |  | 0.429 |
| Never | 9,661 (67.24) | 1,038 (68.79) |  |
| Drinking but less than once a month | 1,113 (7.75) | 107 (7.09) |  |
| Drinking more than once a month | 3,595 (25.02) | 364 (24.12) |  |
| Depressive symptom, n (%) | 5,064 (37.48) | 471 (32.82) | 0.001 |
| Cognitive impairment, n (%) | 1756 (18.43) | 167 (14.67) | 0.002 |
| Physical frailty, n (%) | 878 (7.06) | 65 (6.02) | 0.221 |
| Disability in activities of daily living, n (%) | 2,087 (14.57) | 176 (11.69) | 0.003 |
| Hypertension, n (%) | 5,469 (38.19) | 591 (39.35) | 0.394 |
| Diabetes mellitus, n (%) | 1,957 (13.72) | 203 (13.62) | 0.938 |
| Heart disease, n (%) | 1,684 (11.78) | 270 (18.04) | < 0.001 |
| Stroke, n (%) | 319 (2.23) | 40 (2.65) | 0.330 |
| Cancer, n (%) | 148 (1.03) | 14 (0.93) | 0.810 |

^a^ Group comparisons of baseline characteristics were compared using the Mann-Whitney *U* test for continuous variables and the *χ^2^*-test for categorical variables.

**Supplementary Table S2**. The number of study participants in various social activities.

| Types of social participation | No. of participants (%) |
| --- | --- |
| 1. Interacted with friends | 5567 (35.05%) |
| 1. Played Ma-jong, played chess, played cards, or went to community club | 2920 (18.38%) |
| 1. Provided help to family, friends, or neighbours who do not live with you and who did not pay you for the help | 1073 (6.76%) |
| (4) Went to a sport, social, or other kind of club | 1033 (6.50%) |
| (5) Took part in a community-related organization | 237 (1.49%) |
| (6) Done voluntary or charity work | 100 (0.63%) |
| (7) Cared for a sick or disabled adult who does not live with you and who did not pay you for the help | 120 (0.76%) |
| (8) Attended an educational or training course | 60 (0.38%) |
| (9) Stock investment | 88 (0.55%) |
| (10) Used the Internet | 430 (2.71%) |
| (11) engaged in other activities | 125 (0.79%) |

**Supplementary Table S3**. Description of assignment of covariate characteristics.

| Covariate | Variable types | Encoding for assignment |
| --- | --- | --- |
| Age (years) | Continuous | -- |
| Sex | Categorical | 0 = Female; 1 = Male |
| Place of residence | Categorical | 0 = Village; 1 = Urban |
| Educational attainment | Categorical | 0 = Primary school and lower; 1 = Junior middle school and higher |
| Marital status | Categorical | 0 = Married; 1 = Other (Separated, divorced, widowed and never married) |
| Annual household living expenditure (RMB) | Continuous | -- |
| Smoking status | Categorical | 0 = Never smoking; 1 = Former smoking；2 = Current smoking |
| Alcohol consumption status | Categorical | 0 = Never drinking; 1 = Drinking but less than once a month；2 = Drinking more than once a month |
| Depressive symptom | Categorical | 0 = No；1 = Yes |
| Cognitive function | Categorical | 0 = Intact；1 = Impairment |
| Physical frailty | Categorical | 0 = No；1 = Yes |
| Disability in activities of daily living | Categorical | 0 = No；1 = Yes |
| Hypertension | Categorical | 0 = No；1 = Yes |
| Diabetes mellitus | Categorical | 0 = No；1 = Yes |
| Heart disease | Categorical | 0 = No；1 = Yes |
| Stroke | Categorical | 0 = No；1 = Yes |
| Cancer | Categorical | 0 = No；1 = Yes |

**Supplementary Table S4**. The prospective association between social participation (continuous) and all-cause mortality.

| Exposure status | *HR* (95% *CI*) | | |
| --- | --- | --- | --- |
|  | Model 0 ^a^ | Model 1^b^ | Model 2 ^c^ |
| **Social participation (Continuous)** | 0.79 (0.75, 0.84) *** | 0.85 (0.81, 0.90) *** | 0.89 (0.82, 0.96) ** |
| **Age, years** | -- | 1.10 (1.10, 1.11) *** | 1.08 (1.07, 1.09) *** |
| **Sex** |  |  |  |
| Male | -- | 1.00 (reference) | 1.00 (reference) |
| Female | -- | 0.62 (0.57, 0.67) *** | 0.54 (0.45, 0.64) *** |
| **Place of residence** |  |  |  |
| Village | -- | -- | 1.00 (reference) |
| Urban | -- | -- | 1.08 (0.92, 1.28) |
| **Educational attainment** |  |  |  |
| Primary school and lower | -- | -- | 1.00 (reference) |
| Junior middle school and higher | -- | -- | 0.84 (0.72, 0.99) * |
| **Marital status** |  |  |  |
| Married | -- | -- | 1.00 (reference) |
| Other ^d^ | -- | -- | 1.42 (1.20, 1.66) *** |
| **Annual household living expenditure, RMB** | -- | -- | 1.00 (1.00, 1.00) |
| **Smoking status** |  |  |  |
| Never | -- | -- | 1.00 (reference) |
| Formal | -- | -- | 1.54 (1.26, 1.88) *** |
| Current | -- | -- | 1.40 (1.19, 1.66) *** |
| **Alcohol consumption status** |  |  |  |
| Never | -- | -- | 1.00 (reference) |
| Drinking but less than once a month | -- | -- | 0.74 (0.57, 0.98) * |
| Drinking more than once a month | -- | -- | 1.03 (0.88, 1.19) |
| **Depressive symptom** |  |  |  |
| No | -- | -- | 1.00 (reference) |
| Yes | -- | -- | 1.01 (0.88, 1.16) |
| **Cognitive function** |  |  |  |
| Intact | -- | -- | 1.00 (reference) |
| Impairment | -- | -- | 1.15 (0.99, 1.34) |
| **Physical frailty** |  |  |  |
| No | -- | -- | 1.00 (reference) |
| Yes | -- | -- | 1.71 (1.41, 2.08) *** |
| **Disability in activities of daily living** |  |  |  |
| No | -- | -- | 1.00 (reference) |
| Yes | -- | -- | 1.57 (1.34, 1.84) *** |
| **Hypertension** |  |  |  |
| No | -- | -- | 1.00 (reference) |
| Yes | -- | -- | 1.33 (1.16, 1.51) *** |
| **Diabetes mellitus** |  |  |  |
| No | -- | -- | 1.00 (reference) |
| Yes | -- | -- | 1.27 (1.08, 1.49) ** |
| **Heart disease** |  |  |  |
| No | -- | -- | 1.00 (reference) |
| Yes | -- | -- | 1.26 (1.06, 1.50) ** |
| **Stroke** |  |  |  |
| No | -- | -- | 1.00 (reference) |
| Yes | -- | -- | 0.84 (0.59, 1.21) |
| **Cancer** |  |  |  |
| No | -- | -- | 1.00 (reference) |
| Yes | -- | -- | 2.03 (1.25, 3.29) ** |

^a^ Model 0 was not adjusted for any covariate. ^b^ Model 1 adjusted only for age and sex. ^c^ Model 2 further adjusts for the place of residence, marital status, educational attainment, annual household living expenditures, smoking status, alcohol consumption status, depressive symptom, cognitive function, physical frailty, disability in activities of daily living, hypertension, diabetes mellitus, heart disease, stroke, and cancer based on Model 1. ^d^ Other includes separated, divorced, widowed, and never married. ^*^ *P* ≤ 0.05; ^**^ *P* ≤ 0.01; ^***^ *P* ≤ 0.001. Abbreviations: *HR* = Hazard ratio, *CI* = Confidence interval.

**Supplementary Table S5**. The prospective association between social participation (dichotomy) and all-cause mortality.

| Exposure status | *HR* (95% *CI*) | | |
| --- | --- | --- | --- |
|  | Model 0 ^a^ | Model 1^b^ | Model 2 ^c^ |
| **Social participation (****Dichotomy)** |  |  |  |
| No | 1.00 (reference) | 1.00 (reference) | 1.00 (reference) |
| Yes | 0.78 (0.72, 0.85) *** | 0.84 (0.77, 0.91) *** | 0.88 (0.78, 0.99) * |
| **Age, years** | -- | 1.10 (1.10, 1.11) *** | 1.08 (1.07, 1.09) *** |
| **Sex** |  |  |  |
| Male | -- | 1.00 (reference) | 1.00 (reference) |
| Female | -- | 0.62 (0.57, 0.68) *** | 0.54 (0.45, 0.64) *** |
| **Place of residence** |  |  |  |
| Village | -- | -- | 1.00 (reference) |
| Urban | -- | -- | 1.07 (0.91, 1.27) |
| **Educational attainment** |  |  |  |
| Primary school and lower | -- | -- | 1.00 (reference) |
| Junior middle school and higher | -- | -- | 0.83 (0.71, 0.98) * |
| **Marital status** |  |  |  |
| Married | -- | -- | 1.00 (reference) |
| Other ^d^ | -- | -- | 1.42 (1.20, 1.66) *** |
| **Annual household living expenditure, RMB** | -- | -- | 1.00 (1.00, 1.00) |
| **Smoking status** |  |  |  |
| Never | -- | -- | 1.00 (reference) |
| Formal | -- | -- | 1.54 (1.25, 1.88) *** |
| Current | -- | -- | 1.40 (1.18, 1.66) *** |
| **Alcohol consumption status** |  |  |  |
| Never | -- | -- | 1.00 (reference) |
| Drinking but less than once a month | -- | -- | 0.73 (0.56, 0.96) * |
| Drinking more than once a month | -- | -- | 1.02 (0.88, 1.19) |
| **Depressive symptom** |  |  |  |
| No | -- | -- | 1.00 (reference) |
| Yes | -- | -- | 1.00 (0.88, 1.16) |
| **Cognitive function** |  |  |  |
| Intact | -- | -- | 1.00 (reference) |
| Impairment | -- | -- | 1.16 (0.99, 1.35) |
| **Physical frailty** |  |  |  |
| No | -- | -- | 1.00 (reference) |
| Yes | -- | -- | 1.72 (1.42, 2.09) *** |
| **Disability in activities of daily living** |  |  |  |
| No | -- | -- | 1.00 (reference) |
| Yes | -- | -- | 1.58 (1.35, 1.86) *** |
| **Hypertension** |  |  |  |
| No | -- | -- | 1.00 (reference) |
| Yes | -- | -- | 1.33 (1.17, 1.51) *** |
| **Diabetes mellitus** |  |  |  |
| No | -- | -- | 1.00 (reference) |
| Yes | -- | -- | 1.27 (1.08, 1.49) ** |
| **Heart disease** |  |  |  |
| No | -- | -- | 1.00 (reference) |
| Yes | -- | -- | 1.26 (1.06, 1.49) ** |
| **Stroke** |  |  |  |
| No | -- | -- | 1.00 (reference) |
| Yes | -- | -- | 0.84 (0.58, 1.20) |
| **Cancer** |  |  |  |
| No | -- | -- | 1.00 (reference) |
| Yes | -- | -- | 2.03 (1.25, 3.30) ** |

^a^ Model 0 was not adjusted for any covariate. ^b^ Model 1 adjusted only for age and sex. ^c^ Model 2 further adjusts for the place of residence, marital status, educational attainment, annual household living expenditures, smoking status, alcohol consumption status, depressive symptom, cognitive function, physical frailty, disability in activities of daily living, hypertension, diabetes mellitus, heart disease, stroke, and cancer based on Model 1. ^d^ Other includes separated, divorced, widowed, and never married. ^*^ *P* ≤ 0.05; ^**^ *P* ≤ 0.01; ^***^ *P* ≤ 0.001. Abbreviations: *HR* = Hazard ratio, *CI* = Confidence interval.

**Supplementary Table S6**. The prospective association between social participation (three categories) and all-cause mortality.

| Exposure status | *HR* (95% *CI*) | | |
| --- | --- | --- | --- |
|  | Model 0 ^a^ | Model 1^b^ | Model 2 ^c^ |
| **Social participation (Three categories)** |  |  |  |
| 0 | 1.00 (reference) | 1.00 (reference) | 1.00 (reference) |
| 1 | 0.90 (0.82, 0.98) * | 0.91 (0.83, 1.00) * | 0.93 (0.81, 1.06) |
| ≥ 2 | 0.55 (0.48, 0.63) *** | 0.68 (0.59, 0.78) *** | 0.77 (0.64, 0.94) ** |
| **Age, years** | -- | 1.10 (1.10, 1.11) *** | 1.08 (1.07, 1.09) *** |
| **Sex** |  |  |  |
| Male | -- | 1.00 (reference) | 1.00 (reference) |
| Female | -- | 0.62 (0.57, 0.67) *** | 0.54 (0.45, 0.64) *** |
| **Place of residence** |  |  |  |
| Village | -- | -- | 1.00 (reference) |
| Urban | -- | -- | 1.08 (0.91, 1.28) |
| **Educational attainment** |  |  |  |
| Primary school and lower | -- | -- | 1.00 (reference) |
| Junior middle school and higher | -- | -- | 0.84 (0.71, 0.98) * |
| **Marital status** |  |  |  |
| Married | -- | -- | 1.00 (reference) |
| Other ^d^ | -- | -- | 1.41 (1.20, 1.66) *** |
| **Annual household living expenditure, RMB** | -- | -- | 1.00 (1.00, 1.00) |
| **Smoking status** |  |  |  |
| Never | -- | -- | 1.00 (reference) |
| Formal | -- | -- | 1.54 (1.26, 1.88) *** |
| Current | -- | -- | 1.41 (1.20, 1.67) *** |
| **Alcohol consumption status** |  |  |  |
| Never | -- | -- | 1.00 (reference) |
| Drinking but less than once a month | -- | -- | 0.74 (0.56, 0.97) * |
| Drinking more than once a month | -- | -- | 1.03 (0.88, 1.19) |
| **Depressive symptom** |  |  |  |
| No | -- | -- | 1.00 (reference) |
| Yes | -- | -- | 1.01 (0.88, 1.16) |
| **Cognitive function** |  |  |  |
| Intact | -- | -- | 1.00 (reference) |
| Impairment | -- | -- | 1.15 (0.99, 1.34) |
| **Physical frailty** |  |  |  |
| No | -- | -- | 1.00 (reference) |
| Yes | -- | -- | 1.72 (1.41, 2.08) *** |
| **Disability in activities of daily living** |  |  |  |
| No | -- | -- | 1.00 (reference) |
| Yes | -- | -- | 1.57 (1.34, 1.84) *** |
| **Hypertension** |  |  |  |
| No | -- | -- | 1.00 (reference) |
| Yes | -- | -- | 1.33 (1.16, 1.51) *** |
| **Diabetes mellitus** |  |  |  |
| No | -- | -- | 1.00 (reference) |
| Yes | -- | -- | 1.28 (1.08, 1.49) ** |
| **Heart disease** |  |  |  |
| No | -- | -- | 1.00 (reference) |
| Yes | -- | -- | 1.26 (1.06, 1.50) ** |
| **Stroke** |  |  |  |
| No | -- | -- | 1.00 (reference) |
| Yes | -- | -- | 0.84 (0.59, 1.20) |
| **Cancer** |  |  |  |
| No | -- | -- | 1.00 (reference) |
| Yes | -- | -- | 2.04 (1.26, 3.31) ** |

^a^ Model 0 was not adjusted for any covariate. ^b^ Model 1 adjusted only for age and sex. ^c^ Model 2 further adjusts for the place of residence, marital status, educational attainment, annual household living expenditures, smoking status, alcohol consumption status, depressive symptom, cognitive function, physical frailty, disability in activities of daily living, hypertension, diabetes mellitus, heart disease, stroke, and cancer based on Model 1. ^d^ Other includes separated, divorced, widowed, and never married. ^*^ *P* ≤ 0.05; ^**^ *P* ≤ 0.01; ^***^ *P* ≤ 0.001. Abbreviations: *HR* = Hazard ratio, *CI* = Confidence interval.

**Supplementary Table S7**. Sensitivity analysis.

| Exposure status | Cases/person-years | *HR* (95% *CI*) | | |
| --- | --- | --- | --- | --- |
|  |  | Model 0 ^a^ | Model 1 ^b^ | Model 2 ^c^ |
| **Sensitivity analysis 1** ^d^ |  |  |  |  |
| Social participation (Dichotomy) |  |  |  |  |
| No | 1,053/63,209 | 1.00 (reference) | 1.00 (reference) | 1.00 (reference) |
| Yes | 863/65,132 | 0.79 (0.72, 0.87) *** | 0.85 (0.78, 0.93) *** | 0.86 (0.75, 0.98) * |
| Social participation (Three categories) |  |  |  |  |
| 0 | 1,053/63,209 | 1.00 (reference) | 1.00 (reference) | 1.00 (reference) |
| 1 | 653/43,048 | 0.91 (0.82, 1.01) | 0.92 (0.83, 1.01) | 0.90 (0.78, 1.04) |
| ≥ 2 | 211/22,084 | 0.57 (0.49, 0.66) *** | 0.69 (0.60, 0.80) *** | 0.77 (0.62, 0.94) ** |
| *P trend* | -- | < 0.001 | < 0.001 | 0.011 |
| Social participation (Continuous) | 1,916/128,341 | 0.81 (0.76, 0.85) *** | 0.86 (0.82, 0.92) *** | 0.88 (0.81, 0.96) ** |
| **Sensitivity analysis 2** ^e^ |  |  |  |  |
| Social participation (Dichotomy) |  |  |  |  |
| No | 1,205/63,443 | 1.00 (reference) | 1.00 (reference) | 1.00 (reference) |
| Yes | 970/65,346 | 0.79 (0.72, 0.87) *** | 0.85 (0.78, 0.93) *** | 0.91 (0.83, 0.99) * |
| Social participation (Three categories) |  |  |  |  |
| 0 | 1,205/63,443 | 1.00 (reference) | 1.00 (reference) | 1.00 (reference) |
| 1 | 737/43,187 | 0.91 (0.82, 1.01) | 0.92 (0.83, 1.01) | 0.96 (0.87, 1.06) |
| ≥ 2 | 233/22,159 | 0.57 (0.49, 0.66) *** | 0.69 (0.60, 0.80) *** | 0.78 (0.67, 0.91) ** |
| *P trend* | -- | < 0.001 | < 0.001 | 0.003 |
| Social participation (Continuous) | 2,175/128,789 | 0.81 (0.76, 0.85) *** | 0.86 (0.82, 0.92) *** | 0.91 (0.86, 0.96) ** |

^a^ Model 0 was not adjusted for any covariate. ^b^ Model 1 adjusted only for age and sex. ^c^ Model 2 further adjusts for place of residence, marital status, educational attainment, annual household living expenditures, smoking status, alcohol consumption status, depressive symptom, cognitive function, physical frailty, disability in activities of daily living, hypertension, diabetes mellitus, heart disease, stroke, and cancer based on Model 1. ^d^ Sensitivity analyses 1 were performed after excluding participants with < 2 years of follow-up. ^e^ Sensitivity analyses 2 were performed after multiple imputations of the chained equations. ^*^ *P* ≤ 0.05; ^**^ *P* ≤ 0.01; ^***^ *P* ≤ 0.001. Abbreviations: *HR* = Hazard ratio, *CI* = Confidence interval.
